# Supplementary material for: Patient preferences for diagnostic imaging services: Decentralize or not?
Source: PLoS One. 2025 May 16;20(5):e0301404. doi: 10.1371/journal.pone.0301404 (PMC12084043; doi:10.1371/journal.pone.0301404)
Supplement: Appendix 1 — Scenario introduction. (DOCX) [file pone.0301404.s001.docx]

**Appendix 1**

**Scenario Introduction**

The University of Vermont is studying how to improve access to diagnostic services and which aspects of service delivery patients value. For the purpose of this study, suppose you hurt your arm and your primary care provider wants to send you for an x-ray. You have three options of locations where you can have your imaging done.

In the next section, you will answer 14 choice questions, each with 3 different options regarding where to get your diagnostic services.

When you are asked to choose between locations, assume that everything else about the locations is identical, and that they only differ in the ways described below:

Interpreting Doctor Specialty. Doctors who interpret X-Rays can have different levels of training (specialization). The two levels of training you will be presented with on this survey include:

**General Radiologist** – This means that your images would be interpreted by a Radiologist, which is a doctor who is a specialist in interpreting multiple types of images but does not have specific additional training in the type of image you’re getting. (For example, the doctor interprets X-Rays but also interprets CT scans, ultrasounds, and other images of multiple body systems)

**Specialty Radiologist** – This means that your images would be interpreted by a Radiologist who has additional training interpreting the type of image you’re getting. (for example, the doctor interpreting your arm X-Ray is specialized in reading images of broken bones in the arm).

Primary Care Recommendation. This means that your primary care provider or personal doctor told you about this clinic and told you something positive or specially recommended it. Clinics that are not "recommended" are not necessarily worse than those which are, they just were not specially recommended by your doctor. The options you will be presented with include:

**Yes** – This means that the clinic is recommended by your primary care provider

**No** – This means that the clinic is not specifically recommended by your primary care provider

Wait Time To Results. This means how long it takes to get the results from your x-ray once you have gotten the imaging done, depending on how busy your clinic is. The options you will be presented with include:

$statusquo_x_timeresult_less25 Minutes – This would mean that the clinic will take $statusquo_x_timeresult_less25 minutes to give you the results from your x-ray

$statusquo_x_timeresult Minutes – This would mean that the clinic will take $statusquo_x_timeresult minutes to give you the results from your x-ray

$statusquo_x_timeresult_add25 Minutes – This would mean that the clinic will take $statusquo_x_timeresult_add25 minutes to give you the results from your x-ray

Cost. This means the amount that you have to pay of your own money for the x-ray. The options you will be presented with include:

$$statusquo_x_cost_less25 – This would mean that the clinic will charge $$statusquo_x_cost_less25 for your your x-ray

$$statusquo_x_cost – This would mean that the clinic will charge $$statusquo_x_cost for your your x-ray

$$statusquo_x_cost_add25 – This would mean that the clinic will charge $$statusquo_x_cost_add25 for your your x-ray

Travel Time. Regardless of type of transportation, this would be the maximum time you would need to get to the location for your x-ray services. The options you will be presented with include:

$statusquo_x_timetravel_less25 Minutes – This would mean that it takes you $statusquo_x_timetravel_less25 minutes to travel from your home to the clinic (regardless of the type of transportation)

$statusquo_x_timetravel Minutes – This would mean that it takes you $statusquo_x_timetravel minutes to travel from your home to the clinic (regardless of the type of transportation)

$statusquo_x_timetravel_add25 Minutes – This would mean that it takes you $statusquo_x_timetravel_add25 minutes to travel from your home to the clinic (regardless of the type of transportation)

Wait Time To Appointment. This means how long you would wait in the waiting room, depending on how busy your clinic is. The options you will be presented with include:

$statusquo_x_timewait_less25 Minutes – This would mean that the clinic will take $statusquo_x_timewait_less25 minutes to see you for your x-ray

$statusquo_x_timewait Minutes – This would mean that the clinic will take $statusquo_x_timewait minutes to see you for your x-ray

$statusquo_x_timewait_add25 Minutes – This would mean that the clinic will take $statusquo_x_timewait_add25 minutes to see you for your x-ray

Parking Cost. This is whether or not the parking is free at the clinic. The options you will be presented with include:

Free – This means that the clinic is free to park at

Paid – This means that you would need to pay to park your car at the clinic

Parking Accessibility. This is how easy it is to get to the clinic door from the parking lot, or from the nearest public transit stop. The options you will be presented with include:

$statusquo_x_parkingaccess_less25 Minutes – This would mean that it takes you $statusquo_x_parkingaccess_less25 minutes to travel from your vehicle or bus stop to the clinic waiting room

$statusquo_x_parkingaccess Minutes – This would mean that it takes you $statusquo_x_parkingaccess minutes to travel from your vehicle or bus stop to the clinic waiting room

$statusquo_x_parkingaccess_add25 Minutes – This would mean that it takes you $statusquo_x_parkingaccess_add25 minutes to travel from your vehicle or bus stop to the clinic waiting room

Service. This means the level of satisfaction patients have with the staff's sensitivity to their needs while at the clinic, on a scale of 1 (dissatisfied) to 5 (very satisfied). The options you will be presented with include:

$statusquo_x_service_less1 – This would mean that the clinic is rated $statusquo_x_service_less1 for patient experience, safety, and the quality of your care.

$statusquo_x_service – This would mean that the clinic is rated $statusquo_x_service for patient experience, safety, and the quality of your care.

$statusquo_x_service_plus1 – This would mean that the clinic is rated $statusquo_x_service_plus1 for patient experience, safety, and the quality of your care.

Online Scheduling. This is whether or not the clinic allows you to schedule an appointment yourself online. The options you will be presented with include:

**Not Available** – This means that online scheduling is not available at the clinic

**Available** – This means that online scheduling is available at the clinic

An example of a choice task you could be asked to complete is below, please select the clinic you would most prefer.
