# Supplementary material for: Patient preferences for diagnostic imaging services: Decentralize or not?
Source: PLoS One. 2025 May 16;20(5):e0301404. doi: 10.1371/journal.pone.0301404 (PMC12084043; doi:10.1371/journal.pone.0301404)
Supplement: Appendix 2 — Demographics participants. (DOCX) [file pone.0301404.s002.docx]

| Appendix 2 - Demographic characteristics of participants | | | | | | |
| --- | --- | --- | --- | --- | --- | --- |
|  | **Focus Group 1** | | **Focus Group 2** | | **Overall** | |
|  | N | % | N | % | N | % |
| **Gender** |  |  |  |  |  |  |
| Female | 4 | 0.80 | 4 | 0.57 | 8 | 0.67 |
| Male | 1 | 0.20 | 3 | 0.43 | 4 | 0.33 |
| **Age** |  |  |  |  |  |  |
| 35-44 | 1 | 0.20 | 1 | 0.14 | 2 | 0.17 |
| 45-54 | 1 | 0.20 | 0 | 0.00 | 1 | 0.08 |
| 55-64 | 0 | 0.00 | 3 | 0.43 | 3 | 0.25 |
| 65-74 | 3 | 0.60 | 2 | 0.29 | 5 | 0.42 |
| 75+ | 0 | 0.00 | 1 | 0.14 | 1 | 0.08 |
| **Education***** |  |  |  |  |  |  |
| Bachelor's degree (BA, AB, BS, BBA) | 1 | 0.20 | 2 | 0.29 | 3 | 0.25 |
| Master's degree, Professional, Doctoral degree | 3 | 0.60 | 4 | 0.57 | 7 | 0.58 |
| Some college credit, associate’s degree, no degree | 1 | 0.20 | 1 | 0.14 | 2 | 0.17 |
| **Income** |  |  |  |  |  |  |
| $25,001-$50,000 | 1 | 0.20 | 0 | 0.00 | 1 | 0.08 |
| $50,001-$75,000 | 0 | 0.00 | 1 | 0.14 | 1 | 0.08 |
| $75,001-$100,000 | 2 | 0.40 | 4 | 0.57 | 6 | 0.50 |
| $100,001 or more | 2 | 0.40 | 2 | 0.29 | 4 | 0.33 |
| **Region** |  |  |  |  |  |  |
| Rural | 2 | 0.40 | 1 | 0.14 | 3 | 0.25 |
| Suburban | 3 | 0.60 | 6 | 0.86 | 9 | 0.75 |

*X^2^ tests of independence showed no significant difference between focus groups exists based on gender, age, education, income, or region*

*Opening question*

- *What motivated you to become a ______ (provider role)?*

*Theme questions*

- *As a provider, what do you think of the radiology services at _______ (UVHN facility)?*
- *What influence does UVHN have on your care delivery?*
  - *What influence does it have on your job satisfaction?*
- *What are the most important factors to delivering high-quality and low-cost radiology services? (suggest if they can’t think of any)*
  - *Online scheduling*
  - *Doctor recommendation*
  - *Specialist reading*
  - *Face to face contact for results*
  - *Wait time to be seen*
  - *Travel time*
  - *Time to results*
- *How do you think a patient might answer that question?*
- *How can services be improved at _______ (UVHN facility)?*
- *What do you think when you hear ‘decentralized outpatient radiology’?*
