# Supplementary material for: Patient preferences for diagnostic imaging services: Decentralize or not?
Source: PLoS One. 2025 May 16;20(5):e0301404. doi: 10.1371/journal.pone.0301404 (PMC12084043; doi:10.1371/journal.pone.0301404)
Supplement: Appendix 3 — Survey text. (PDF) [file pone.0301404.s003.pdf]

Research Information

Title of Study: Understanding Patient Preferences for Decentralized Imaging Service Offerings in the United States

Principal Investigator (PI): Eline van den Broek-Altenburg, PhD

Funder: Department of Radiology, University of Vermont Larner College of Medicine

Introduction
You are being invited to take part in this research study as a survey panel member for Centiment, Inc. This study is being conducted by Eline van den Broek-Altenburg at the University of Vermont.

Purpose
This study aims to better understand the choices people make when deciding where to go for non-urgent diagnostic imaging (such as x-rays and MRIs), in order to improve the care offered to patients.

Study Procedures
If you take part in the study, you will be asked to fill out a survey with some basic information about yourself (age, gender, income, health status, etc.), after which you will be presented with 14 choice scenarios. In these choice scenarios, you will be told what type of care you are in need of, and given three clinic locations to choose from to get care. The clinics will vary in a number of ways (distance, price, specialization, etc.). In total, the survey and 14 choice tasks will take approximately 20 minutes to complete.

Benefits
As a participant in this research study, there may not be direct benefit for you; however, information from this study may benefit other people now or in the future.

Risks
To protect your confidentiality, we will not collect any information that will identify you.

Costs
There will be no costs to you for participation in this research study.

Compensation
For taking part in this research study, you may be reimbursed for your time and inconvenience, at the rates set by your panel company. If you have concerns or questions about reimbursement, please contact the relevant company.

Confidentiality
All information collected about you during the course of this study will be stored without any identifiers. No one will be able to match you to your answers.

Voluntary Participation/Withdrawal
Taking part in this study is voluntary. You are free to not answer any questions or withdraw at any time. You may choose not to take part in this study, or if you decide to take part, you can change your mind and withdraw from the study. As your information is full de-identified at the time of collection, should you choose not to participate after submission, your data cannot be withdrawn and will be used.

Single Choice

q1_consent

Please review the certification statement below:

Answers

By checking this box I certify that I have read the consent document which has been presented to me. By doing so, I will be considered a subject in this study, have been informed of its aims, and am aware that I can withdraw at any time.

I do not wish to participate.

2.

Demographics

Single Choice

q2_gender

What gender identity best describes you?

Answers

Man

Woman

Non-Binary

Prefer not to say

Prefer to Self Describe

Last label is 'Other'

Question Display Condition

Answer Display Conditions

Randomise

Optional

Validation

Layout:

1 column

Segment

male

Display Condition

$q2_gender eq 1

Segment

female

Display Condition

$q2_gender eq 2

Text Question

q3_age

What is your age, in years?

Question Display Condition

Optional

Validation :

(($q3_age=~/^[0-9]+$/) && ($q3_age>=18))

Error Text:

Only numbers allowed! (Must be over age of 18)

Dimensions

Segment

age_18_24

Display Condition

$q3_age>=18 && $q3_age<=24

Segment

age_25_34

Display Condition

$q3_age>=25 && $q3_age<=34

Segment

age_35_44

Display Condition

$q3_age>=35 && $q3_age<=44

Segment

age_45_54

Display Condition

$q3_age>=45 && $q3_age<=54

Segment

age_55_64

Display Condition

$q3_age>=55 && $q3_age<=64

Segment

age_65plus

Display Condition

$q3_age>=65

Single Choice

q4_ethnicity

Please select the option below that best represents your ethnic heritage

Answers

Not Hispanic or Latino or Spanish Origin

Hispanic or Latino or Spanish Origin

Multiple Choice

q5_race

Which race do you most closely identify with?

Answers

White

Black

Asian

Native Hawaiian or Other Pacific Islander

American Indian or Alaska Native

Last label is 'Other'

Last label is 'None'

Question Display Condition

Answer Display Conditions

Randomise

Optional

Validation

Layout:

2 column

Single Choice

q6_education

What is the highest degree or level of school you have completed? If currently enrolled, highest degree already received.

Answers

Less than High School

High school graduate, diploma or the equivalent

Some college credit, no degree

Associate degree: occupational, tech, vocational

Associate degree: academic program

Bachelor's degree (BA, AB, BS, BBA)

Master's degree

Professional, Doctoral degree

Last label is 'Other'

Question Display Condition

Answer Display Conditions

Randomise

Optional

Validation

Layout:

Dropdown

Text Question

q7_income

What is your annual income (approximate), in USD? (e.g., 44000)

Question Display Condition

Optional

Validation :

(($q7_income=~/^[0-9]+$/) && ($q7_income>=0))

Error Text:

Numbers greater than 0 only

Dimensions

Single Choice

q8_rural

Which of the following best describes the area you live in?

Answers

Urban

Suburban

Rural

Single Choice

q9_insurance

Please select the option below that best describes your health insurance status

Answers

Insured - Private (Blue Cross Blue Shield, Aetna, etc.)

Insured - Medicare (adults 65+)

Insured - Medicaid (low-income adults)

Uninsured

Single Choice

q10_deductible

If you have an annual deductible, have you already met it this year?

Answers

Yes

No

I do not have a deductible

Single Choice

q11_residency

Do you live in the State of Vermont?

Answers

Yes

No

Segment

all_completes

Display Condition

$q3_age >= 18 && $q11_residency eq 1

Segment

screenout_age

Display Condition

$q3_age < 18

Segment

screenout_residency

Display Condition

$q11_residency eq 2

Branch

23. Screen Out

Display Condition

$q3_age < 18 || $q11_residency eq 2

Segment

xray

Display Condition

$group eq 1

Branch

3. Status Quo Determination - Xray

Display Condition

$group eq 1

Segment

mri

Display Condition

$group eq 2

Branch

4. Status Quo Determination - MRI

Display Condition

$group eq 2

3.

Status Quo Determination - Xray

Text

Next, we want to ask you a couple of questions about your most recent visit for an X-Ray.

If you have not had one before, answer the below questions with your best guess based on your experiences with your current doctor and hospital.

Single Choice

sq_xray_specialty

If you were to get an x-ray at your usual clinic, do you think it would be interpreted (read) by a specially trained radiologist, specific to your treatment? (E.g., a radiologist who specializes in arm injuries)

Answers

Yes

Unsure, likely yes

Unsure, likely no

No

Last label is 'Other'

Question Display Condition

Answer Display Conditions

Randomise

Optional

Validation

Layout:

Rating

Derived

statusquo_x_specialty

if ((($sq_xray_specialty) eq 1)|(($sq_xray_specialty) eq 2)) {return "Specialty Radiologist"} else {return "General Radiologist"}

Single Choice

sq_xray_pcprec

Would the clinic you get your x-ray at be recommended to you by your primary care physician?

Answers

Yes

No

Derived

statusquo_x_pcprec

if ((($sq_xray_pcprec) eq 1)) {return "Yes"} else {return "No"}

Text Question

sq_xray_timeresult

Most people wait between 15 and 120 minutes for the results of an x-ray. Please enter the length of time in that range you think you would wait for your results (in minutes):

Question Display Condition

Optional

Validation :

($sq_xray_timeresult=~/^[0-9]\d*$/) && ($sq_xray_timeresult>=15) && ($sq_xray_timeresult<=120)

Error Text:

Please check to make sure the answer you gave is between the two numbers in the question above.

Dimensions

Derived

statusquo_x_timeresult

int($sq_xray_timeresult)

Derived

statusquo_x_timeresult_add25

int($sq_xray_timeresult*1.25)

Derived

statusquo_x_timeresult_less25

if ((int($sq_xray_timeresult*0.75))<1) {return "0"} else {return (int($sq_xray_timeresult*0.75))}

Text Question

sq_xray_cost

Most people pay between $10 and $300 out of pocket for an x-ray, depending on their insurance. Please enter the amount in that range you think you would pay for an x-ray (in dollars):

Question Display Condition

Optional

Validation :

($sq_xray_cost=~/^[0-9]\d*$/) && ($sq_xray_cost>=10) && ($sq_xray_cost<=300)

Error Text:

Please check to make sure the answer you gave is between the two numbers in the question above.

Dimensions

Derived

statusquo_x_cost

int($sq_xray_cost)

Derived

statusquo_x_cost_add25

int($sq_xray_cost*1.25)

Derived

statusquo_x_cost_less25

if ((int($sq_xray_cost*0.75))<1) {return "0"} else {return (int($sq_xray_cost*0.75))}

Text Question

sq_xray_timetravel

It takes most people between 5 and 120 minutes to travel to their clinic for an x-ray. Please enter the length of time in that range you think you would travel for your x-ray (in minutes):

Question Display Condition

Optional

Validation :

($sq_xray_timewait=~/^[0-9]\d*$/) && ($sq_xray_timewait>=5) && ($sq_xray_timewait<=120)

Error Text:

Please check to make sure the answer you gave is between the two numbers in the question above.

Dimensions

Derived

statusquo_x_timetravel

int($sq_xray_timetravel)

Derived

statusquo_x_timetravel_add25

int($sq_xray_timetravel*1.25)

Derived

statusquo_x_timetravel_less25

if ((int($sq_xray_timetravel*0.75))<1) {return "0"} else {return (int($sq_xray_timetravel*0.75))}

Text Question

sq_xray_timewait

Most people wait between 5 and 45 minutes to be seen for an x-ray. Please enter the length of time in that range you think you would wait for your x-ray (in minutes):

Question Display Condition

Optional

Validation :

($sq_xray_timewait=~/^[0-9]\d*$/) && ($sq_xray_timewait>=5) && ($sq_xray_timewait<=45)

Error Text:

Please check to make sure the answer you gave is between the two numbers in the question above.

Dimensions

Derived

statusquo_x_timewait

int($sq_xray_timewait)

Derived

statusquo_x_timewait_add25

int($sq_xray_timewait*1.25)

Derived

statusquo_x_timewait_less25

if ((int($sq_xray_timewait*0.75))<1) {return "0"} else {return (int($sq_xray_timewait*0.75))}

Single Choice

sq_xray_parking

At your usual clinic, is there free parking?

Answers

Yes

No

Derived

statusquo_x_freeparking

if ((($sq_xray_parking) eq 1)) {return "Free"} else {return "Paid"}

Text Question

sq_xray_parkingaccess

It takes most people between 2 and 20 minutes to get from their car / bus stop to the x-ray waiting room. Please enter the length of time in that range you think it normally takes you (in minutes):

Question Display Condition

Optional

Validation :

($sq_xray_parkingaccess=~/^[0-9]\d*$/) && ($sq_xray_parkingaccess>=2) && ($sq_xray_parkingaccess<=20)

Error Text:

Please check to make sure the answer you gave is between the two numbers in the question above.

Dimensions

Derived

statusquo_x_parkingaccess

int($sq_xray_timewait)

Derived

statusquo_x_parkingaccess_add25

int($sq_xray_timewait)*1.25

Derived

statusquo_x_parkingaccess_less25

int($sq_xray_timewait)*0.75

Single Choice

sq_xray_service

At your usual x-ray clinic, how would you rate the service, considering your experience, safety, and the quality of your care?

Answers

1 Star (Very Poor)

2 Stars (Poor)

3 Stars (Adequate)

4 Stars (Good)

5 Stars (Very Good)

Last label is 'Other'

Question Display Condition

Answer Display Conditions

Randomise

Optional

Validation

Layout:

Rating

Derived

statusquo_x_service

$sq_xray_service." Star: ".("*" x int($sq_xray_service))

Derived

statusquo_x_service_plus1

if (($sq_xray_service) eq 5) {return("3 Star: ***")} else {return((int($sq_xray_service)+1)." Star: ".("*" x (int($sq_xray_service)+1)))}

Derived

statusquo_x_service_less1

if (($sq_xray_service) eq 1) {return("3 Star: ***")} else {return((int($sq_xray_service)-1)." Star: ".("*" x (int($sq_xray_service)-1)))}

Single Choice

sq_xray_onlinesch

At your usual clinic, do you have the option to schedule your appointment online?

Answers

Yes

No

Derived

statusquo_x_onlinesch

if ((($sq_xray_onlinesch) eq 1)) {return "Available"} else {return "Not Available"}

Branch

5. Scenario Introduction

Display Condition

$group eq 1

4.

Status Quo Determination - MRI

Text

Next, we want to ask you a couple of questions about your most recent visit for an MRI.

If you have not had one before, answer the below questions with your best guess based on your experiences with your current doctor and hospital.

Single Choice

sq_mri_specialty

If you were to get an MRI at your usual clinic, do you think it would be interpreted (read) by a specially trained radiologist, specific to your treatment? (E.g., a radiologist who specializes in arm injuries)

Answers

Yes

Unsure, likely yes

Unsure, likely no

No

Last label is 'Other'

Question Display Condition

Answer Display Conditions

Randomise

Optional

Validation

Layout:

Rating

Derived

statusquo_m_specialty

if ((($sq_mri_specialty) eq 1)|(($sq_mri_specialty) eq 2)) {return "Specialty Radiologist"} else {return "General Radiologist"}

Single Choice

sq_mri_pcprec

Would the clinic you get your MRI at be recommended to you by your primary care physician?

Answers

Yes

No

Derived

statusquo_m_pcprec

if ((($sq_mri_pcprec) eq 1)) {return "Yes"} else {return "No"}

Text Question

sq_mri_timeresult

Most people wait between 2 and 72 hours for the results of an MRI. Please enter the length of time in that range you think you would wait for your results (in hours):

Question Display Condition

Optional

Validation :

($sq_mri_timeresult=~/^[0-9]\d*$/) && ($sq_mri_timeresult>=2) && ($sq_mri_timeresult<=72)

Error Text:

Please check to make sure the answer you gave is between the two numbers in the question above.

Dimensions

Derived

statusquo_m_timeresult

int($sq_mri_timeresult)

Derived

statusquo_m_timeresult_add25

int($sq_mri_timeresult*1.25)

Derived

statusquo_m_timeresult_less25

if ((int($sq_mri_timeresult*0.75))<1) {return "0"} else {return (int($sq_mri_timeresult*0.75))}

Text Question

sq_mri_cost

Most people pay between $10 and $2,000 out of pocket for an MRI, depending on their insurance. Please enter the amount in that range you think you would pay for an MRI (in dollars):

Question Display Condition

Optional

Validation :

($sq_mri_cost=~/^[0-9]\d*$/) && ($sq_mri_cost>=10) && ($sq_mri_cost<=2000)

Error Text:

Please check to make sure the answer you gave is between the two numbers in the question above.

Dimensions

Derived

statusquo_m_cost

int($sq_mri_cost)

Derived

statusquo_m_cost_add25

int($sq_mri_cost*1.25)

Derived

statusquo_m_cost_less25

if ((int($sq_mri_cost*0.75))<1) {return "0"} else {return (int($sq_mri_cost*0.75))}

Text Question

sq_mri_timetravel

It takes most people between 5 and 120 minutes to travel to their clinic for an MRI. Please enter the length of time in that range you think you would travel for your MRI (in minutes):

Question Display Condition

Optional

Validation :

($sq_mri_timetravel=~/^[0-9]\d*$/) && ($sq_mri_timetravel>=5) && ($sq_mri_timetravel<=120)

Error Text:

Please check to make sure the answer you gave is between the two numbers in the question above.

Dimensions

Derived

statusquo_m_timetravel

int($sq_mri_timetravel)

Derived

statusquo_m_timetravel_add25

int($sq_mri_timetravel*1.25)

Derived

statusquo_m_timetravel_less25

if ((int($sq_mri_timetravel*0.75))<1) {return "0"} else {return (int($sq_mri_timetravel*0.75))}

Text Question

sq_mri_timewait

Most people wait between 3 and 28 days to be seen for an MRI. Please enter the length of time in that range you think you would wait for your MRI (in days):

Question Display Condition

Optional

Validation :

($sq_mri_timewait=~/^[0-9]\d*$/) && ($sq_mri_timewait>=3) && ($sq_mri_timewait<=28)

Error Text:

Please check to make sure the answer you gave is between the two numbers in the question above.

Dimensions

Derived

statusquo_m_timewait

int($sq_mri_timewait)

Derived

statusquo_m_timewait_add25

int($sq_mri_timewait*1.25)

Derived

statusquo_m_timewait_less25

if ((int($sq_mri_timewait*0.75))<1) {return "0"} else {return (int($sq_mri_timewait*0.75))}

Single Choice

sq_mri_parking

At your usual clinic, is there free parking?

Answers

Yes

No

Derived

statusquo_m_freeparking

if ((($sq_mri_parking) eq 1)) {return "Free"} else {return "Paid"}

Text Question

sq_mri_parkingaccess

It takes most people between 2 and 20 minutes to get from their car / bus stop to the MRI waiting room. Please enter the length of time in that range you think it normally takes you (in minutes):

Question Display Condition

Optional

Validation :

($sq_mri_parkingaccess=~/^[0-9]\d*$/) && ($sq_mri_parkingaccess>=2) && ($sq_mri_parkingaccess<=20)

Error Text:

Please check to make sure the answer you gave is between the two numbers in the question above.

Dimensions

Derived

statusquo_m_parkingaccess

int($sq_mri_parkingaccess)

Derived

statusquo_m_parkingaccess_add25

int($sq_mri_parkingaccess)*1.25

Derived

statusquo_m_parkingaccess_less25

int($sq_mri_parkingaccess)*0.75

Single Choice

sq_mri_service

At your usual MRI clinic, how would you rate the service, considering your experience, safety, and the quality of your care?

Answers

1 Star (Very Poor)

2 Stars (Poor)

3 Stars (Adequate)

4 Stars (Good)

5 Stars (Very Good)

Last label is 'Other'

Question Display Condition

Answer Display Conditions

Randomise

Optional

Validation

Layout:

Rating

Derived

statusquo_m_service

$sq_mri_service." Star: ".("*" x int($sq_mri_service))

Derived

statusquo_m_service_plus1

if (($sq_mri_service) eq 5) {return("3 Star: ***")} else {return((int($sq_mri_service)+1)." Star: ".("*" x (int($sq_mri_service)+1)))}

Derived

statusquo_m_service_less1

if (($sq_mri_service) eq 1) {return("3 Star: ***")} else {return((int($sq_mri_service)-1)." Star: ".("*" x (int($sq_mri_service)-1)))}

Single Choice

sq_mri_onlinesch

At your usual clinic, do you have the option to schedule your appointment online?

Answers

Yes

No

Derived

statusquo_m_onlinesch

if ((($sq_mri_onlinesch) eq 1)) {return "Available"} else {return "Not Available"}

5.

Scenario Introduction

Text

The University of Vermont is studying how to improve access to diagnostic services and which aspects of service delivery patients value. For the purpose of this study, suppose you

hurt your arm and your primary care provider wants to send you for an x-ray. You have three options of locations where you can have your imaging done.

In the next section, you will answer 14 choice questions, each with 3 different options regarding where to get your diagnostic services.

When you are asked to choose between locations, assume that everything else about the locations is identical, and that they only differ in the ways described below:

Interpreting Doctor Specialty. Doctors who interpret X-Rays can have different levels of training (specialization). The two levels of training you will be presented with on this survey include:

General Radiologist – This means that your images would be interpreted by a Radiologist, which is a doctor who is a specialist in interpreting multiple types of images but does not have specific additional training in the type of image you're getting. (For example, the doctor interprets X-Rays but also interprets CT scans, ultrasounds, and other images of multiple body systems)

Specialty Radiologist – This means that your images would be interpreted by a Radiologist who has additional training interpreting the type of image you're getting. (for example, the doctor interpreting your arm X-Ray is specialized in reading images of broken bones in the arm).

Primary Care Recommendation. This means that your primary care provider or personal doctor told you about this clinic and told you something positive or specially recommended it. Clinics that are not "recommended" are not necessarily worse than those which are, they just were not specially recommended by your doctor. The options you will be presented with include:

Yes – This means that the clinic is recommended by your primary care provider

No – This means that the clinic is not specifically recommended by your primary care provider

Wait Time To Results. This means how long it takes to get the results from your x-ray once you have gotten the imaging done, depending on how busy your clinic is. The options you will be presented with include:

$statusquo_x_timeresult_less25 Minutes – This would mean that the clinic will take $statusquo_x_timeresult_less25 minutes to give you the results from your x-ray

$statusquo_x_timeresult Minutes – This would mean that the clinic will take $statusquo_x_timeresult minutes to give you the results from your x-ray

$statusquo_x_timeresult_add25 Minutes – This would mean that the clinic will take $statusquo_x_timeresult_add25 minutes to give you the results from your x-ray

Cost. This means the amount that you have to pay of your own money for the x-ray. The options you will be presented with include:

$$statusquo_x_cost_less25 – This would mean that the clinic will charge $$statusquo_x_cost_less25 for your your x-ray

$$statusquo_x_cost – This would mean that the clinic will charge $$statusquo_x_cost for your your x-ray

$$statusquo_x_cost_add25 – This would mean that the clinic will charge $$statusquo_x_cost_add25 for your your x-ray

Travel Time. Regardless of type of transportation, this would be the maximum time you would need to get to the location for your x-ray services. The options you will be presented with include:

$statusquo_x_timetravel_less25 Minutes – This would mean that it takes you $statusquo_x_timetravel_less25 minutes to travel from your home to the clinic (regardless of the type of transportation)

$statusquo_x_timetravel Minutes – This would mean that it takes you $statusquo_x_timetravel minutes to travel from your home to the clinic (regardless of the type of transportation)

$statusquo_x_timetravel_add25 Minutes – This would mean that it takes you $statusquo_x_timetravel_add25 minutes to travel from your home to the clinic (regardless of the type of transportation)

Wait Time To Appointment. This means how long you would wait in the waiting room, depending on how busy your clinic is. The options you will be presented with include:

$statusquo_x_timewait_less25 Minutes – This would mean that the clinic will take $statusquo_x_timewait_less25 minutes to see you for your x-ray

$statusquo_x_timewait Minutes – This would mean that the clinic will take $statusquo_x_timewait minutes to see you for your x-ray

$statusquo_x_timewait_add25 Minutes – This would mean that the clinic will take $statusquo_x_timewait_add25 minutes to see you for your x-ray

Parking Cost. This is whether or not the parking is free at the clinic. The options you will be presented with include:

Free – This means that the clinic is free to park at

Paid – This means that you would need to pay to park your car at the clinic

Parking Accessibility. This is how easy it is to get to the clinic door from the parking lot, or from the nearest public transit stop. The options you will be presented with include:

$statusquo_x_parkingaccess_less25 Minutes – This would mean that it takes you $statusquo_x_parkingaccess_less25 minutes to travel from your vehicle or bus stop to the clinic waiting room

$statusquo_x_parkingaccess Minutes – This would mean that it takes you $statusquo_x_parkingaccess minutes to travel from your vehicle or bus stop to the clinic waiting room

$statusquo_x_parkingaccess_add25 Minutes – This would mean that it takes you $statusquo_x_parkingaccess_add25 minutes to travel from your vehicle or bus stop to the clinic waiting room

Service. This means the level of satisfaction patients have with the staff's sensitivity to their needs while at the clinic, on a scale of 1 (dissatisfied) to 5 (very satisfied). The options you will be presented with include:

$statusquo_x_service_less1 – This would mean that the clinic is rated $statusquo_x_service_less1 for patient experience, safety, and the quality of your care.

$statusquo_x_service – This would mean that the clinic is rated $statusquo_x_service for patient experience, safety, and the quality of your care.

$statusquo_x_service_plus1 – This would mean that the clinic is rated $statusquo_x_service_plus1 for patient experience, safety, and the quality of your care.

Online Scheduling. This is whether or not the clinic allows you to schedule an appointment yourself online. The options you will be presented with include:

Not Available – This means that online scheduling is not available at the clinic

Available – This means that online scheduling is available at the clinic

An example of a choice task you could be asked to complete is below, please select the clinic you would most prefer.

Display Condition

Text

The University of Vermont is studying how to improve access to diagnostic services and which aspects of service delivery patients value. For the purpose of this study, suppose you hurt your arm a while ago, and are having persistent pain in the area. Your primary care provider wants to send you for an MRI. You have three options of locations where you can have your imaging done.

In the next section, you will answer 14 choice questions, each with 3 different options regarding where to get your diagnostic services.

When you are asked to choose between locations, assume that everything else about the locations is identical, and that they only differ in the ways described below:

Interpreting Doctor Specialty. Doctors who interpret MRIs can have different levels of training (specialization). The two levels of training you will be presented with on this survey include:

General Radiologist – This means that your images would be interpreted by a Radiologist, which is a doctor who is a specialist in interpreting multiple types of images but does not have specific additional training in the type of image you're getting. (For example, the doctor interprets MRIs but also interprets CT scans, ultrasounds, and other images of multiple body systems)

Specialty Radiologist – This means that your images would be interpreted by a Radiologist who has additional training interpreting the type of image you're getting. (for example, the doctor interpreting your arm MRI is specialized in reading images of muscles and bones in the arm).

Primary Care Recommendation. This means that your primary care provider or personal doctor told you about this clinic and told you something positive or specially recommended it. Clinics that are not "recommended" are not necessarily worse than those which are, they just were not specially recommended by your doctor. The options you will be presented with include:

Yes – This means that the clinic is recommended by your primary care provider

No – This means that the clinic is not specifically recommended by your primary care provider

Wait Time To Results. This means how long it takes to get the results from your MRI once you have gotten the imaging done, depending on how busy your clinic is. The options you will be presented with include:

$statusquo_m_timeresult_less25 Hours– This would mean that the clinic will take $statusquo_m_timeresult_less25 hours to give you the results from your MRI

$statusquo_m_timeresult Hours – This would mean that the clinic will take $statusquo_m_timeresult hours to give you the results from your MRI

$statusquo_m_timeresult_add25 Hours – This would mean that the clinic will take $statusquo_m_timeresult_add25 hours to give you the results from your MRI

Cost. This means the amount that you have to pay of your own money for the MRI. The options you will be presented with include:

$$statusquo_m_cost_less25 – This would mean that the clinic will charge $$statusquo_m_cost_less25 for your your MRI

$$statusquo_m_cost – This would mean that the clinic will charge $$statusquo_m_cost for your your MRI

$$statusquo_m_cost_add25 – This would mean that the clinic will charge $$statusquo_m_cost_add25 for your your MRI

Travel Time. Regardless of type of transportation, this would be the maximum time you would need to get to the location for your MRI services. The options you will be presented with include:

$statusquo_m_timetravel_less25 Minutes – This would mean that it takes you $statusquo_m_timetravel_less25 minutes to travel from your home to the clinic (regardless of the type of transportation)

$statusquo_m_timetravel Minutes – This would mean that it takes you $statusquo_m_timetravel minutes to travel from your home to the clinic (regardless of the type of transportation)

$statusquo_m_timetravel_add25 Minutes – This would mean that it takes you $statusquo_m_timetravel_add25 minutes to travel from your home to the clinic (regardless of the type of transportation)

Wait Time To Appointment. This means how long it takes to get an appointment for your MRI, depending on how busy your clinic is. The options you will be presented with include:

$statusquo_m_timeresult_less25 Days – This would mean that the clinic will take $statusquo_m_timeresult_less25 days to get you in for your MRI

$statusquo_m_timeresult Days – This would mean that the clinic will take $statusquo_m_timeresult days to get you in for your MRI

$statusquo_m_timeresult_add25 Days – This would mean that the clinic will take $statusquo_m_timeresult_add25 days to get you in for your MRI

Parking Cost. This is whether or not the parking is free at the clinic. The options you will be presented with include:

Free – This means that the clinic is free to park at

Paid – This means that you would need to pay to park your car at the clinic

Parking Accessibility. This is how easy it is to get to the clinic door from the parking lot, or from the nearest public transit stop. The options you will be presented with include:

$statusquo_m_parkingaccess_less25 Minutes – This would mean that it takes you $statusquo_m_parkingaccess_less25 minutes to travel from your vehicle or bus stop to the clinic waiting room

$statusquo_m_parkingaccess Minutes – This would mean that it takes you $statusquo_m_parkingaccess minutes to travel from your vehicle or bus stop to the clinic waiting room

$statusquo_m_parkingaccess_add25 Minutes – This would mean that it takes you $statusquo_m_parkingaccess_add25 minutes to travel from your vehicle or bus stop to the clinic waiting room

Service. This means the level of satisfaction patients have with the staff's sensitivity to their needs while at the clinic, on a scale of 1 (dissatisfied) to 5 (very satisfied). The options you will be presented with include:

$statusquo_m_service_less1 – This would mean that the clinic is rated $statusquo_m_service_less1 for patient experience, safety, and the quality of your care.

$statusquo_m_service – This would mean that the clinic is rated $statusquo_m_service for patient experience, safety, and the quality of your care.

$statusquo_m_service_plus1 – This would mean that the clinic is rated $statusquo_m_service_plus1 for patient experience, safety, and the quality of your care.

Online Scheduling. This is whether or not the clinic allows you to schedule an appointment yourself online. The options you will be presented with include:

Not Available – This means that online scheduling is not available at the clinic

Available – This means that online scheduling is available at the clinic

An example of a choice task you could be asked to complete is below, please select the clinic you would most prefer.

Display Condition

$group eq 2

Experiment

X-Ray

random design row

Display Condition

$group eq 1

Experiment

MRI

random design row

Display Condition

$group eq 2

Single Choice

q8_understanding

Do you feel that you understand what you will be asked to do on the next screen?

Answers

Yes

No

Segment

screenout_understanding

Display Condition

$q8_understanding eq 2

Branch

23. Screen Out

Display Condition

$q8_understanding eq 2

6.

Choice Task 1

Experiment

X-Ray

random design row

Display Condition

$group eq 1

Experiment

MRI

random design row

Display Condition

$group eq 2

7.

Choice Task 2

Experiment

X-Ray

random design row

Display Condition

$group eq 1

Experiment

MRI

random design row

Display Condition

$group eq 2

8.

Choice Task 3

Experiment

X-Ray

random design row

Display Condition

$group eq 1

Experiment

MRI

random design row

Display Condition

$group eq 2

9.

Choice Task 4

Experiment

X-Ray

random design row

Display Condition

$group eq 1

Experiment

MRI

random design row

Display Condition

$group eq 2

10.

Choice Task 5

Experiment

X-Ray

random design row

Display Condition

$group eq 1

Experiment

MRI

random design row

Display Condition

$group eq 2

11.

Choice Task 6

Experiment

X-Ray

random design row

Display Condition

$group eq 1

Experiment

MRI

random design row

Display Condition

$group eq 2

12.

Choice Task 7

Experiment

X-Ray

random design row

Display Condition

$group eq 1

Experiment

MRI

random design row

Display Condition

$group eq 2

13.

Halfway there!

Text Question

attention_check

You are halfway through the choice questions-- great job! To continue, please enter the correct answer below (numbers only):

What is 1 + 3?

Question Display Condition

Optional

Validation :

$attention_check==4

Error Text:

Incorrect, please check your math.

Dimensions

14.

Choice Task 8

Experiment

X-Ray

random design row

Display Condition

$group eq 1

Experiment

MRI

random design row

Display Condition

$group eq 2

15.

Choice Task 9

Experiment

X-Ray

random design row

Display Condition

$group eq 1

Experiment

MRI

random design row

Display Condition

$group eq 2

16.

Choice Task 10

Experiment

X-Ray

random design row

Display Condition

$group eq 1

Experiment

MRI

random design row

Display Condition

$group eq 2

17.

Choice Task 11

Experiment

X-Ray

random design row

Display Condition

$group eq 1

Experiment

MRI

random design row

Display Condition

$group eq 2

18.

Choice Task 12

Experiment

X-Ray

random design row

Display Condition

$group eq 1

Experiment

MRI

random design row

Display Condition

$group eq 2

19.

Choice Task 13

Experiment

X-Ray

random design row

Display Condition

$group eq 1

Experiment

MRI

random design row

Display Condition

$group eq 2

20.

Choice Task 14

Experiment

X-Ray

random design row

Display Condition

$group eq 1

Experiment

MRI

random design row

Display Condition

$group eq 2

21.

Closeout Questions

Matrix of Single Choice

importance

Please rank the importance of the various factors you used to make your decisions, from most important, to least important:

Row Questions

Cost

Primary Care Recommendation

Parking Cost

Parking Accessibility

Wait Time to Appointment

Time to Results

Travel Time to Clinic

Service Rating

Interpreting Doctor Specialty

Online Scheduling Availability

Answers

Most Important (1)

2

3

4

5

6

7

8

9

Least Important (10)

Question Display Condition

Row Display Conditions

Answer Display Conditions

Randomise

Optional

Validation

Repeat Column Heading

Long Question layout

Layout:

Drag'n Drop

Multiple Choice

ignored

Please select any factors which you IGNORED in making your decisions:

Answers

Cost

Primary Care Recommendation

Parking Cost

Parking Accessibility

Wait Time to Appointment

Time to Results

Travel Time to Clinic

Service Rating

Interpreting Doctor Specialty

Online Scheduling Availability

None (I did not ignore any of the above)

2nd-Last label is 'Other'

Last label is 'None'

Question Display Condition

Answer Display Conditions

Randomise

Optional

Validation

Layout:

2 column

Matrix of Single Choice

attitudinals

Please rank how much you agree with the following statements:

Row Questions

"I do not need health insurance."

"Health insurance is not worth the money it costs."

"I am more likely to take risks than the average person."

"I can overcome illness without help from a medically trained person."

"I am healthier than the average person."

Answers

Strongly Disagree

Disagree

Uncertain

Agree

Strongly Agree

Single Choice

chroniccond

Do you have a chronic condition that requires regular care from a doctor?

Answers

No

Yes

Single Choice

num_xrays

How many x-rays have you had in your lifetime?

Answers

0

1

2

3

4

5 or more

Last label is 'Other'

Question Display Condition

$group eq 1

Answer Display Conditions

Randomise

Optional

Validation

Layout:

Dropdown

Single Choice

num_mris

How many MRIs have you had in your lifetime?

Answers

0

1

2

3

4

5 or more

Last label is 'Other'

Question Display Condition

$group eq 2

Answer Display Conditions

Randomise

Optional

Validation

Layout:

Dropdown

Single Choice

difficulty

How difficult did you find this survey?

Answers

Not at all

A little bit

Somewhat Challenging

Difficult

Very Difficult

Last label is 'Other'

Question Display Condition

Answer Display Conditions

Randomise

Optional

Validation

Layout:

Rating

Text Question

comments

Do you have any comments on the survey, or your answers?

Question Display Condition

Optional

Validation

Dimensions

22.

End Page

Text

Thank You

That concludes the survey. Thank you for your participation, you are helping to improve our understanding of Vermont's healthcare system.

Please remember to click "Submit" to finish your survey so that you can be compensated for your time!

Exit

Complete

23.

Screen Out

Text

You have selected that you do not wish to participate, or continue, in this survey experiment, or you have selected a disqualifying answer. Your participation is entirely voluntary, and you have the right to exit at any time. If you have reached this page in error, please close this browser window and navigate to the link you were sent originally to re-start. If you have any remaining questions or concerns, you can reach out to the Principal Investigator Eline van den Broek-Altenburg at Eline.Altenburg@med.uvm.edu, or the University of Vermont Research Protections Office at (802) 656-5040.

Please click "Submit" to complete your participation.

Exit

Screen out
