# Supplementary material for: Patient preferences for diagnostic imaging services: Decentralize or not?
Source: PLoS One. 2025 May 16;20(5):e0301404. doi: 10.1371/journal.pone.0301404 (PMC12084043; doi:10.1371/journal.pone.0301404)
Supplement: Attachment — Submitted filename: Response to reviewers_decentralized imaging.docx [file pone.0301404.s005.docx]

1. Please ensure that your manuscript meets PLOS ONE's style requirements, including those for file naming. The PLOS ONE style templates can be found at

<https://journals.plos.org/plosone/s/file?id=wjVg/PLOSOne_formatting_sample_main_body.pdf> and<https://journals.plos.org/plosone/s/file?id=ba62/PLOSOne_formatting_sample_title_authors_affiliations.pdf>

We edited the manuscript and title page according to the PLOS ONE style templates.

2. Please provide additional details regarding participant consent. In the ethics statement in the Methods and online submission information, please ensure that you have specified (1) whether consent was informed and (2) what type you obtained (for instance, written or verbal, and if verbal, how it was documented and witnessed). If your study included minors, state whether you obtained consent from parents or guardians. If the need for consent was waived by the ethics committee, please include this information.

We added to the methods and online submission that consent was informed through a written statement at the start of the online survey. Without the online consent, respondents were not able to proceed. The answers were recorded in the data.

If you are reporting a retrospective study of medical records or archived samples, please ensure that you have discussed whether all data were fully anonymized before you accessed them and/or whether the IRB or ethics committee waived the requirement for informed consent. If patients provided informed written consent to have data from their medical records used in research, please include this information.

We have noted this in the Methods section of the manuscript.

3. . We note that the grant information you provided in the ‘Funding Information’ and ‘Financial Disclosure’ sections do not match.

When you resubmit, please ensure that you provide the correct grant numbers for the awards you received for your study in the ‘Funding Information’ section.

We matched the funding information with financial disclosure. We added the appropriate awards number.

4. Please provide a complete Data Availability Statement in the submission form, ensuring you include all necessary access information or a reason for why you are unable to make your data freely accessible. If your research concerns only data provided within your submission, please write "All data are in the manuscript and/or supporting information files" as your Data Availability Statement.

We provided a completed Data Availability Statement in the submission form. We have now shared our data from this study to a public repository as well as with a supporting information file with this submission. The public repository can be found here: <https://github.com/ElinevandenBroek/>DecentralizedImaging

5. Please include a separate caption for each figure in your manuscript.

We do include a separate caption for each figure.

**Response to Reviewers**

**Reviewer #1**: This is an interesting and important topic given the rising demand. However, the manuscript needs major revisions to be publishable.

We very much appreciate your feedback and respond to each point raised below.

Abstract: p2 l44: It is unnecessary to specify the abbreviation again, as HORD has already been defined.

Thank you, we deleted second mention at p2 l44.

Introduction: p3 l72: The reference needs formatting. This seems to be mistaken.

Thank you, we formatted the reference.

Introduction: p4 l88: The abbreviation CMS needs to be defined.

We added Centers for Medicare and Medicaid Services.

Methods: p5 l119-123: This paragraph should be placed in the experimental design section since some of the information given is repeated in that section. Please consider restructuring/rewriting. Nonetheless, the authors need to give more detail on the literature research (where? when? search terms?) as well as the mentioned pilot study. What were the aims? Who was interviewed, etc.? Are there any references on the pilot and literature?

We completely rewrote the methods section to include a lot more detail about the experimental design, the aim of the study, the participants and the pilot data. We also moved the results of the qualitative study to the results section.

Methods: p6 l144: Until now, the reader has no idea which attributes will be used. The qualitative as well as pilot study have been described without any details. The manuscript clearly lacks results of the qualitative study parts.

In the new text, the paragraph in the Methods section now reads as:” We conducted focus groups among patients at a middle-size academic medical center who volunteered to share their experiences with imaging services. The results of that research are reported in the results section. Following the analysis of the focus groups, we included the following attributes: whether the interpreting radiologist is a general or sub-specialty radiologist; whether the clinic was recommended by their primary care physician (PCP); time to results; out of pocket cost; wait time to an appointment; travel time to the clinic; parking costs; parking accessibility; service; and whether or not online scheduling is available. Service is a multifactorial attribute (e.g., staff attentiveness and facility amenities) combined into a star rating. The rating scale is between one and five stars, with a five-star rating representing an excellent service and a one-star rating suggesting a poor service, as rated by other hypothetical patients.”

We deliberately moved the results to the Results section, including the results of the focus groups. It is now clear in the Methods section how the DCE was constructed and the Results section clarifies the specific findings.

Experimental design: How many levels per attribute were used? The manuscript would benefit from a table showing attributes and levels. What about the actual design? Was it a full design? Which software was used to create the design?

The text now also clarifies: “Each choice task had three different clinics which varied by 10 different attributes, which were determined by prior qualitative research. Four attributes had 2 levels and six attributes had 3 levels.”

We clarified in the text that we used the software NGene to design an experimental design. We also included a table with all the attributes and levels.

Data source: p7 l189: Which consistency checks were used? What was the bot-behavior check, and how did the authors check for failing attention? This needs to be clarified.

Bots were identified by the RegEx program and manual review of the free text entry boxes. Typically, the bots in our sample entered nonsense or repeated the question’s text into those fields. We deleted respondents with w=either missing,"1","6","dsfvrg rfg", "mroe ogod", "the best", "very easy" in their answers.

For attention, we then filtered by completion time over a threshold and also filtered any respondents out that showed straight-lining behavior.

We added this clarification to the text.

Statistical Methods & Latent Class Analysis: Why are these separate paragraphs? The information given is quite repetitive. Please be more precise.

For our analytic approach, we used mixed logit models and latent class analysis. LCA was a sub-paragraph of statistical methods. We took out the subtitle and checked for repetition.

Table 2: p11: Please define your attributes. Without an understanding of how the attributes (and levels?) were defined and described for the participants, it is not possible to conclude anything from this table. Crucial information is missing, which is a major deficit of the manuscript.

We added a Table in the Methods section with all the attributes and levels to provide an understanding of how the attributes and levels were defined.

Latent Class Analysis: p12: How many analyses were performed? Did you just perform the two-class model or 3-, 4-, and 5-class models as well? What was the rationale for choosing the two classes? It seems too easy to just present the two-class option without discussing other options or at least reasons for rejecting different solutions.

We did compare the results of a 3-class model; 4-class model and 5-class model and found the best model fit for the 2-class model, based on the log likelihood and Bayesian Information Criteria (BIC). We explained this in the text.

Attribute importance: p15: Do you have any ideas on why the results of the rating are not completely equal to the DCE?

These are not equal to the DCE since in our models, we estimate relative utilities. We added text to this paragraph to clarify.

Discussion: p16: The discussion is rather short and superficial. To what extent may the university setting have influenced the results? Might the results look different for a rural area with no university medical center nearby?

We added text to the discussion section to address these questions.

Limitations: p16: I think a major limitation is the inclusion of only one university medical center. Due to that, the representativeness of the results must be questioned.

We agree with the reviewer that we should be conservative with regard to out-of-sample predictions. We therefore added some text to the limitations sections and also checked the Discussion section for consistency.

General: The manuscript has too many repetitions and should be rewritten/restructured to be more precise.

We have completely rewritten the manuscript and restructured some sections.

Reviewer #2: The aim of this paper is to employ a discrete choice experiment (DCE) to determine individuals' preferences for outpatient diagnostic imaging services. This inquiry is particularly pertinent given that the rising demand for such technologies may necessitate increased availability, and the way to move forward must depend on people’s preferences. The chosen methodology is appropriate, as a DCE is effective in ranking preferences for various attributes and levels, as well as in calculating the willingness to pay for those specific attributes. Additionally, techniques like latent class analysis within a DCE can be used to explore how preferences vary across different demographic groups, which can aid in the more tailored design of services to better meet the needs of the populations they are intended to serve.

Although the paper generally employs sound methodologies, there are several critical issues that need to be addressed. The most significant of these concerns is the insufficient explanation of the methodology and design, which is particularly vital in a DCE. Additionally, the paper lacks a cohesive narrative and clear policy implications. Below, I will outline my comments in the order that these issues appear in the document:

Abstract:

Please, do not use acronyms in the abstract.

We have specified all acronyms with full names but since we do use them repeatedly throughout the abstract.

Introduction:

Line 72, page 3: “(…) back on the same patient within a 3-week period 5.” – Is the 5 a typo?

Thank you, yes, this was supposed to be a subscript to refer to the reference which we have now fixed.

Lines 78-80, page 3: “Some hospital systems are now looking at options to develop their own RRCs to provide convenient and efficient locations, without sacrificing the technological or interpretation quality, while leveraging the existing trust patients may have with their hospital system” – Any reference to support this claim?

We added the relevant references to the claim.

Lines 80-82, page 3: “This is a potentially valuable innovation for patients, as they would be able to receive the “specialty read” quality at locations with shorter wait times and lower out-of-pocket costs.” – In my opinion this is a problem regarding the narrative of this paper. What the paper ends up saying is that patients want the traditional service (which is superior), but faster and cheaper. I don’t think this is enough as a policy implication for this paper, since there is no need for a DCE to know this.

The background of this study is that a midsize academic medical center in the Northeast considered building a HORD and decentralizing some services away from the hospital. Partly this was due to commercial competition with pop-up imaging services and partly because of the challenge the pop-ups bring for necessary secondary reads in the tertiary care center. From our qualitative work, we know that many patients do want the traditional service but end up going to pop-up services (for example, from the back of a car or truck) because it is cheaper and more accessible. So, when organizing a decentralized service from the hospital, the main question was not so much what attributes of the service patients care about, but the tradeoffs they make when they decide where to get these services they need. The marginal rates of substitution are important to inform the business model of a new to establish decentralized clinic. This is why we decided to do a DCE and elicit patient preferences and study preference heterogeneity. The hope was to predict uptake based on different levels of attributes.

Lines 91-96, page 4: “Existing research on patient preferences in diagnostic imaging services is sparse, however, and what exists is often contradictory 9-15. Though survey studies have explored from whom patients prefer to learn their results and how (e.g., by phone, e-mail, or in-person), findings to date have been inconsistent 9–11,16. For example, one study found a majority preference for learning results in-person and from a radiologist10 while another found that most patients prefer to learn results from their primary care provider, over the phone.” – Is this all? This seems like a very limited discussion of the literature. Can you please provide more depth to this description of the literature?

We have added text to substantiate the various references in this paragraph ads we cite 7 studies. It now reads:

Existing research on patient preferences in diagnostic imaging services is sparse, however, and what exists is often contradictory ^9-15^. One study looked at patient preferences for CT and MRI imaging results and focused on how, from whom, and how soon patients preferred the results ^10^. The authors used a survey and asked those questions directly of patients. They found that patients wanted their results communicated much sooner than is currently practiced. Optimizing patient satisfaction may require a new communication model. This study just focused on these aspects of the services, however, and the authors were not able to assess trade-offs that patients make from the survey data. Another study focused receiving radiology results and reviewing the images and findings directly with a radiologist after completion of an examination ^11^. Patients prefer hearing examination results from both their ordering provider and the interpreting radiologist. These results were consistent with another study focused on results communication ^12^. These are interesting findings, however, only focus on results and not preferences for where to seek services. Another study focused on the referral mechanism ^13^ and the importance of wait time for test results ^14^. None of these studies focused on describing which aspects of imaging services were important and preferred by patients, which has bene done in other fields such as preferences for primary care services ^15^ and preferences for health service delivery in general ^16^.

Methods:

Line 152, page 6: “Situation 1): ”For the purpose of this study, suppose you hurt your arm (…)” – Is there a reason why it is an “arm” for the x-ray and “back” fort the MRI.

We looked at most common reasons for acute X-ray which included fractures. Arm fractures are the most common. For MRI, one of the most common uses is with spinal injuries or low back pain.

We added “based on common reasons for imaging services” to the manuscript text.

Line 153, page 6: “You have three options (…)” – Is there a reason why you chose 3 alternatives instead of the traditional 2 alternatives in a DCE? Is there any concern that 3 alternatives and 14 choice sets may be too much? Any discussion on potential cognitive burden?

There is not a “traditional” number of alternatives in a DCE. We create an efficient design described in the methods section where we need a combination of minimum sample size, limited number of choice tasks and alternatives/levels. With the 3-alternatives design we get more information about the utility weights that respondents assign to different attributes with optimizing the number of choice tasks.

Line 162, page 6: “These selected attributes included (…)” – Where are the levels? It is impossible to review a DCE paper without the levels. Can you please provide a table with all attributes and respective levels? You also need a lot more detail on how the attributes and (especially) levels were chosen. Also, the example choice set in appendix lacks resolution.

We have added Table 1 which provides an overview of all the attributes and levels and descriptions. We have uploaded a new figure with an example choice task.

Line 182, page 7: “Centiment contacted approximately 472 (…)” – Is it approximately 472 or exactly 472?

They contacted more individuals but only 472 replied to their invitation to participate. We clarified this in the text by deleting “approximately”.

Line 188-190, page 7: “We excluded 98 subjects for failing consistency criteria, qualifying for a closed quota, failing a bot-behavior check, or failing an attention (response quality) check” – Can you provide more detail? What were these consistency criteria or attention checks, for example?

Bots were identified by the RegEx program and manual review of the free text entry boxes. Typically, the bots in our sample entered nonsense or repeated the question’s text into those fields. We deleted respondents with w=either missing,"1","6","dsfvrg rfg", "mroe ogod", "the best", "very easy" in their answers.

For attention, we then filtered by completion time over a threshold and also filtered any respondents out that showed straight-lining behavior.

We added this clarification to the text.

Line 190, page 7: “final sample of 170: 84 in Xray, 86 in MRI.” – Are these enough? I know you have 14 choice sets with 3 alternatives each, but still an unusually low number of observations since you are dividing the analysis into two DCE, with later latent class analysis. Care to comment on what lead you to believe this is enough? You say in line 93, page 8 that “We used NGene 1.2.1 (ChoiceMetrics, 192 2018) to estimate the minimum sample size required for this study.”, but what methodology is this?

A design is considered more efficient if it can produce more efficient data in the sense that more reliable parameter estimates can be achieved with an equal or lower sample size. Where orthogonal designs minimize correlations between the attributes to zero; D-efficient designs are aimed at minimizing (co) variances of parameter estimates; A-efficient designs only looks at variances not covariances; and S-efficient is a sample size efficient design aiming to minimize the sample size needed to obtain statistically significant parameter estimates. These measures are “prior” estimates which can be based on researchers’ prior expectations on what the estimated model parameters will be based on literature or sample data.

We’ve explained the process of our prior estimates in the text and added that we used an S-efficient design to check the minimum sample size.

Line 205-211, page 8 – I would cut this entire paragraph. I don’t think it adds anything to the paper.

We completely rewrote the methods section and this text was deleted.

Results:

Line 222-223, page 9: “receiving the X-ray choice questions had had an average of 4.8 previous images while MRI patients had had 2.9 .” Is this per year?

Ever (as they remember). We clarified in the text.

Line 228, page 10: “Mixed logit Results” – How many iterations did you use in the mixed logit?”

We used 100 iterations. We added this to the text.

Line 249, page 12: “Latent Class Analysis” – The latent class analysis is for which DCE? The Xray or MRI?

This is combining the data for both services.

Line 254, page 12: “We found that 54.7 percent of patients were in class 1 and 45.3 percent in class 2” – Why 2 classes? Which criteria did you use?

We compared the results of a 3-class model; 4-class model and 5-class model and found the best model fit for the 2-class model, based on the log likelihood and Bayesian Information Criteria (BIC).

We added this to the text.

Line 256, page 12: “of being in class 2 (p<0.02) which we therefore labeled as the “Experienced Patients” class” – I think there is a mistake here because in the table the experienced patients are class 1 and not 2. Also, why did you choose the terms “Experienced Patients” and “New Patients”. Not sure the text explains that choice very well.

Thank you for catching that error, we corrected the text. We had a long discussion with clinicians about how to label the classes. “Frequent flyers” seemed to capture class 1 well, but clinicians said that that terms has a negative connotation. We all agreed that “experienced” makes it clear that patients frequently receive this services and are therefore more familiar with attribute options and have defined preferences for these attributes.

Line 269 to 275, page 13 – I think the font is different in this section

We changed the font size.

Line 296-297, page 14 – “$2 more to go to a clinic that was recommended by their PCP; $0.70

more for online scheduling; $1.70 more for a 1-point higher star rating; $0.80 more to have a

clinic that would be 1 minutes closer than their current one; $0.65 more for a clinic that had a

1-hour shorter wait and $0.17 more for a clinic that would decrease their walk-up time by 1

minute. The New Patients were willing to pay $17 more for online scheduling and $4 more for

a higher star rating.” – I find it hard to evaluate the willingness to pay part of the paper without the levels of the out of pocket in the DCE.

Costs were “pivoted” around a respondent’s current out of pocket costs: $25 less or $25 more. We used a pivot style stated choice data for OOP costs to include a reference alternative whose attributes remain invariant across replications for the same respondent. We added the levels and clarifications in Table 1. The magnitude of WTP should therefore be interpreted in terms of “more or less than current”. We clarified in the text.

Discussion:

Line 327, page 15: “others only We termed this group” – typo?

Yes, we corrected (added) the text.how trade-offs are made by individual

Line 337-341, page 16: “This is a concrete message to medical centers seeking to decentralize their services away from the main hospital: patients will want to know that the new location offers the same service level in addition to convenience and will be relying on their primary care providers for advice, suggesting outreach to primary care providers will be important for success.” – This is basically saying that decentralized services must keep the characteristics of the traditional services. But I am not sure this is possible. Therefore, this does not seem like a viable narrative/policy implication. If it was possible to create a better, faster, cheaper, more convenient service than it would have been created. The issue here is that tradeoffs need to be made. So, your question should be, for example, how much cheaper certain services would have to be to compensate for some lack of quality.

Agreed, this is the gist of the analysis. We clarified this in the text and described how our follow-up work will further analyze these trade-offs and preference heterogeneity.

Limitations:

Line 344-349, page 16 - There is so much more to say about limitations. The authors never comment on the sample size or the complexity of the design (14 choice sets with 10 attributes and 3 alternatives is a lot). Certainly, there is something to say about the attributes and levels and how they were chosen, but no details are offered in the text. The authors never show the survey (which should be in appendix). There must be something in the survey and how people may have interpreted things that should be noted in the limitations.

We added sections to the limitations addressing the complex design and propose extensions of the current study to further analyze preferences at the national level. We emphasize that we are not making any out-of-sample predictions but that this study provides a first step in more work in this area.

Conclusion:

Line 356, page 16: “(…) diagnostic imaging services in both acute and more chronic situations (…)” – Is there anything in the paper about acute vs. chronic?

Yes, we clarify in the abstract, methods section, results and limitations that we are taking into account that preferences may look differently in acute situations than in more chronic situations which is why we chose to ask questions regarding seeking an X-ray and MRI.

Line 356-360, page 16-17: “had a strong preference for a HORD that would provide services at lower costs, where their images would be interpreted by a specialty radiologist, the clinic would be recommended by their PCP, online scheduling would be available, service rating were higher, and travel 359 and wait times would be shorter.” – This is just saying they want it all. Who doesn’t? This goes back to my earlier comment that your conclusions end up being that people just want more (and better) of everything. But that is not a new finding. You need to rearrange the narrative of the paper to talk more about trade-offs and how the information you provide on trade-offs can help policy making.

We have rearranged the paper and clarified that it’s the trade-offs that we are interested in when advising on how to design such new decentralized services. We are also interested in patient preference heterogeneity, but we did not have enough data to do this analysis which we will focus on in follow-up work with more data.
