# Supplementary material for: Patient preferences for diagnostic imaging services: Decentralize or not?
Source: PLoS One. 2025 May 16;20(5):e0301404. doi: 10.1371/journal.pone.0301404 (PMC12084043; doi:10.1371/journal.pone.0301404)
Supplement: Attachment — Submitted filename: Response to Reviewers_decentralized imaging_v2.docx [file pone.0301404.s006.docx]

**Response to Reviewers**

**Reviewer #1:** During the revision, the manuscript has improved. The issues raised have been addressed by the authors. However, the manuscript still needs revisions to be publishable. Especially the methods section needs clarification.

Thank you, we very much appreciate your additional comments to improve the manuscript. We respond to each point raised below.

Introduction: p4 l.91-104: This section is not an introduction to me, but discussion. I suggest moving the paragraph to the discussion section.

The intention of this paragraph was to introduce the gap in the literature. In response to this comment the paragraph was rewritten and some of the material was moved to the discussion. We have deleted the sentence “To successfully decentralize imaging services at a larger scale, however, it is important to define value not just from the perspective of the healthcare system, but also from the perspective of the patient.” In the previous paragraph to make clear this is purely a description of the literature.

Methods - general: The whole section needs more references! Authors need to declare the references used. There are numerous pages without any reference in this section. Moreover, the methods section needs restructuring and/or subheadings. Right now, it is confusing since qualitative study parts and the description of the experimental DCE design mix up.

The Methods section largely describes the methods we used for this particular study. We added several references to clarify the analytic approach or refer to others explaining the emthods; the remainder is a description of the current study.

We completely reorganized the structuring of the methods section as well to separate the finding of the qualitative work which informed the DCE design. The methods section now starts with the qualitative research. We hope the new structure takes away any confusion.

Methods: p5 l.117-124: This paragraph is not related to experimental design of the DCE. It is rather an explanation of the underlying problem of preference elicitation in all-day routine. Maybe move to the introduction.

We deleted the first few paragraphs of the Methods section and cite this work that describes stated preferences design in detail.

Methods: p6 l.138-143: This is not related to experimental design of the DCE, but a description of the qualitative study. Please clarify. Nonetheless, the authors need to give more detail on the literature research (where? when? search terms?) as well as the mentioned qualitative research. What were the aims? How were potential attributes defined? Are there any references?

We very much appreciate the comments from the reviewer, as we realized a description of this work was missing in prior versions. We added a detailed description of the qualitative work which the Methods section now starts with.

Methods: p6 l144: Until now, the reader has no idea how attributes were defined. Instead, authors just mention “Four attributes had 2 levels and six attributes had 3 levels “. The qualitative as well as pilot study have been described without any details. The manuscript clearly lacks results of the qualitative study parts. Moreover, I would suggest adding Table 1 directly to the statement on the final number of attributes/levels.

Methods: p6 l145-156: This is not related to experimental design of the DCE, but attribute selection. It should be placed upfront.

After restructuring the methods section and adding detailed information about the qualitative research, the construction of the levels should now be clarified. We appreciate the suggestion to add Table 1 (now Table 2) in the Methods section, so we moved it up.

Experimental design: The manuscript would benefit from more detail on the actual design. Any overlaps used? What about consistency tests?

This paper is written for a broad audience and we tried to not be too specific, although we already included a lot of detail on the design. Per reviewers’ request we added text to the experimental design section including regarding overlaps. Consistency tests are described in the data section.

Methods: p7/8 l190-205: Suggest adding a subheading since these paragraphs deal with survey/questionnaire design rather that experimental design of the DCE

Thank you for the suggestion. We added another subheading here, separating the DCE from the experimental design description. We also added Figure 1 illustrating a choice question.

Data source: p10 l211ff: What is the Centiment panel? Is it open to anybody or linked to a certain institution? More information on the panel would be nice.

We have added text to this section:

“Centiment is a survey company which recruits individuals to answer surveys to generate rewards for themselves or to pledge their earnings to a nonprofit of their choice and it is open to anyone to participate. Centiment has engineered complex systems to manage their respondents and ensure they are providing thoughtful responses.”

Data source: p10 l211ff: Which consistency checks were used? What was the bot-behavior check, and how did the authors check for failing attention? This still needs to be clarified.

Thank you for this comment. Responding to the previous comment about this, we added a clarification to the text:

“Bots were identified by the RegEx program and manual review of the free text entry boxes. Typically, the bots in our sample entered nonsense or repeated the question’s text into those fields. We deleted respondents with w=either missing,"1","6","dsfvrg rfg", "mroe ogod", "the best", "very easy" in their answers.”

We have now done three additional things to clarify:

- We have made the threshold more explicit: “For attention, we then checked for consistency and filtered by completion time over a threshold of 7 minutes” since it is not possible to read and respond to the questions within that period of time.
- We have clarified what straight-lining means: and also filtered any respondents out that showed straight-lining behavior, meaning that a respondent would always pick the same response to the choice questions”. We added three references here.
- We added to the text regarding bot-behavior and clarified which respondents were deleted for suspected bot behavior. We also added: “In addition, we seeked to identify fraudulent data by defining a priori indicators that warranted elimination or suspicion; an approach borrowed from another study 26.” We added a reference to a relevant study here as well to clarify the approach.

Data source: p10 l221: Which quota was used?

The quotas that were agreed on included 5 percent of census on age by decade, gender, and region; 3 percent of census on race/ethnicity; and 50 percent above and below national median income of $65k/year. We added this clarification to the text.

Data source: p11 l232: Authors state that the minimum sample size was estimated using NGene. What was the result?

We added a clarification to the text: “The S-efficient design we generated in NGene showed that we needed a minimum of 55 respondents so our sample size was this was a sufficient”.

Statistical Methods: This section lacks references!

We added several relevant references, including:

30.	Hensher DA, Greene WH. The mixed logit model: the state of practice. Transportation. 2003 May;30:133-76.

31.	McFadden D, Train K. Mixed MNL models for discrete response. J Appl Econ. 2000;15(5):447–70.

32.	Hole AR. Mixed logit modeling in Stata--an overview. InUnited Kingdom Stata Users' Group Meetings 2013 2013 Sep 16 (No. 23). Stata Users Group.

33.	van den Broek-Altenburg EM, Atherly AJ, Hess S, Benson J. The effect of unobserved preferences and race on vaccination hesitancy for COVID-19 vaccines: implications for health disparities. Journal of Managed Care & Specialty Pharmacy. 2021 Sep;27(9-a Suppl):S2-11.

34.	Greene WH, Hensher DA, Rose J. Accounting for heterogeneity in the variance of unobserved effects in mixed logit models. Transport Res Part B Methodol. 2006;40(1):75–92.

35.	van den Broek-Altenburg, E., et al., Exploring Heterogeneity in Moral Terminology in Palliative Care Consultations. BMC palliative care, 2020.

36.	Yoo HI. lclogit2: An enhanced command to fit latent class conditional logit models. The Stata Journal. 2020 Jun;20(2):405-25.

37.	Greene WH, Hensher DA. A latent class model for discrete choice analysis: contrasts with mixed logit. Transportation Research Part B: Methodological. 2003 Sep 1;37(8):681-98.

Qualitative research: p11 l232: As mentioned earlier, some paragraphs from the introduction can be added here

We have moved this text.

Qualitative research: p12 l259: “From the thematic analysis of the focus groups” – Which qualitative method (and/or software) was used? Did authors rely on any theoretical frame? In addition, this section is lacking real results. How many participants? What was their background and how were they recruited?

We added to the text to clarify the number of participants, the software that was used and how they were recruited.

Mixed Logit results:p14: Why was the number of iterations set to 100? Any rationale on that?

Thank you for this question as we realized the typo in the text, 100 should be 1000. The reason we used 1000 draws is that the literature suggests that, depending on the number of random parameters, stable mixed-logit estimation requires at least 1000 draws. We edited and added the justification.

Latent Class Analysis: Thank you. My comments from the first review have all been answered. Well done.

Thank you, we appreciate the comments and feedback.

Limitations: p16: I think a major limitation is the inclusion of only one university medical center. Due to that, the representativeness of the results must be questioned.

A discrete choice experiment / stated preferences research generally has a challenge with external validity since choice questions are asked in a hypothetical setting and study populations are usually not representative of specific choice settings. This is a known problem. For this reason, we had already written: “This study was performed within the hospital service area of a midsize academic medical center in a relatively rural area in the Northeast, so it is unclear how these results translate to patient preferences at the national level.” We have also refrained from making strong translations to a broader/national audience.

To further clarify this limitation, we cite the relevant literature and added:

“External validity remains a challenge for any DCE study even though, while an important component, it has been argued by others that the investigation of external validity should be much broader than a comparison of final outcomes.”

General: The manuscript should be rewritten/restructured to be more precise, especially in the methods section.

We have drastically rewritten en restructured the manuscript to address all the reviewer’s comments. We appreciate their comments.

**Reviewer #2:** I would like to thank the authors for their corrections.

However, I still have a few concerns I would like to see addressed.

Thank you, we very much appreciate your additional comments to improve the manuscript. We respond to each point raised below.

Note that the pages and lines I refer to are the ones in the track changes version.

Page 4, line 113 – “which has bene done in other fields” – typo, correct to “been”.

This word has been deleted in the new version of the manuscript.

Page 5, line 132 – “One option to measure hard to measure choice attributes is to use stated preferences (SP) data” – please avoid using more than two or three acronyms throughout the paper. In this case, you use SP so rarely that it probably didn’t even save more than two or three words.

Agreed. And this sentence has also been deleted from the text of the new version of the manuscript.

Page 8, line 203 to 205 – “In some cases, a study may contain more choice tasks than the researcher wants to ask respondents. In this case, a blocking experimental design can be used.” – I don’t think this sentence is very well written, to the point that it took me several readings to not understand the opposite of what you want to say. The case for using blocks is usually related to the need for a very big design and fear of cognitive burden. In those cases, researchers may split the design into 2 or more blocks so that each person answers a fewer amount of choice sets, avoiding cognitive burden and tiredness and therefore assuring the quality of the answers. I assume you want to say something within these lines, but that was not what was coming through.

Correct, that was the meaning of this sentence. We edited and it now reads: “In some cases, an efficient design includes many choice tasks. In this case, a blocking experimental design can be used to avoid too much of a cognitive burden for the respondent. Blocks are subsets of the choice questions, which are usually equally sized, that contain a limited number of choice questions for each respondent. In those cases, respondents are randomly assigned to a block and answer the choice questions in that block instead of the entire design.”

Page 8, line 208 to 210 – “In our study, respondents were asked 6 questions regarding X-ray services and 6 questions regarding Magnetic Resonance Imaging (MRI) services.” – Here I am confused. First, are these two block of the same design or two different designs? While you are using the same design for both, preferences may differ for each service, therefore, I would think you would have to randomly run the full design separately, which would count as two designs, even if they are equal. Second, is it correct to run the same design for both (e.g., shouldn’t some level values be different depending on the service?). Third, 6+6 is 12, but you say they had 14 choice sets. Where are the other two? Also, page 13, line 294 “134 were assigned to the arm X-ray group, 134 to the back MRI group.” Seems to imply they are just two different groups. I apologise if I completely misinterpreted what you wrote, but as of now, I think this part of the manuscript is not very clear.

You are completely right. Thank you for catching this. The text is wrong since this was a previous iteration of this study but we changed the approach. We have edited the text accordingly: “In our study, respondents were randomized to either 14 choice questions related to X-ray services or 14 questions releated to MR services. The vignette was different for the two DCE’s and some of the levels, such as costs, were also different. “

Page 9 – line 226 – Thank you for adding this table. Since the table in the choice sets needs to be simpler for visual simplicity reasons, I would like to know if respondents received instructions on the attributes and levels. This is way I stress that you should include the survey (or at least important parts of the survey) in appendix, for matters of clarity and for the reviewers to be able to properly evaluate the merits and limitations of the paper. Also, within table one, in the parking attribute, do you specify how much people need to pay in the level where they pay? Also, wouldn’t this be extremely correlated with the costs attribute? I see the point of the parking accessibility, since this implies other levels of stress in trying to park close to the place, but the cost is a little bit harder to understand. What is done is done, but it might deserve a comment from you. Another issue I detected is the Service attribute where I don’t know what these stars mean. Is it one star out of 5? What do they mean? Did respondents have access to this information (or maybe this is obvious to anyone living in the US?).

Thank you, we added the Scenario Introduction as an Appendix (supporting information). Since we created the survey in SurveyEngine, it is challenging to export the entire survey, but it will be available on request. We clarified using SurveyEngine for the online design in the methods section.

Thank you also for your comments about the parking costs and the star level attributes. We added text to clarify both. You could be correct regarding potential correlation between costs and parking. We addressed this in the limitations section: “Regarding the attribute of parking costs: this could be correlated with the cost attribute. We did check for correlation and di not find the two coefficients to be correlated. This could be due to parking being considered a “different kind of expense”, but we would leave out costs for parking out of the design in a next round of data collection.”

We also added text to clarify the star rating system:

“The star ratings are based on the CMS Five-Star Quality Rating System which were created to help consumers, their families, and caregivers compare clinics more easily and to help identify areas about which they may want to ask questions. A rating of 1 or 2 stars means that the clinic’s performance was below the average of other agencies on selected measures; it doesn't necessarily mean care is poor. A rating of 4 or 5 stars means that the clinic’s performance was above the average of other agencies on selected measures”

Page 13, line 299 to 302 – “Bots were identified by the RegEx program and manual review of the free text entry boxes. Typically, the bots in our sample entered nonsense or repeated the question’s text into those fields. We deleted respondents with w=either missing,"1","6","dsfvrg rfg", "mroe ogod", "the best", "very easy" in their answers.” - Sorry, I didn't want you to add this. It was just to be sure what type of attention checks you might have used. For example, some people have included repeated choice sets to try to identify inconsistencies. Some people check the time taken to answer (which you have now included). I was just confirming if you had included any attention check in the survey itself that deserved being referred.

We deleted the sentence “We deleted respondents with w=either missing,"1","6","dsfvrg rfg", "mroe ogod", "the best", "very easy" in their answers.” Yes, we also included an attention check and edited the sentence to clarify:

“For attention, we then checked for consistency and filtered by completion time over a threshold of 7 minutes and also filtered any respondents out that showed straight-lining behavior, meaning that a respondent would always pick the same response to the choice questions 267-298.”

Page 15, line 339 to 342 – “A question guide aimed at understanding how patients perceive their radiology experiences led participants through their focus group session. Questions were intended to be exploratory and were sometimes followed by probes to allow differences between patient insights and experiences to emerge.” – Could you add relevant material related to the focus groups in appendix? For example, you state that you had a list of questions. Could you add those? Also, could you add some description of the participants? Do you think they were somewhat representative. I know you won’t have fully representative participants in a focus group, but were you able to have some diversity (gender, age, ethnicity, social background, etc…)

We have completely restructured the Methods section to add a lot more information about the qualitative research. We added a table with the attributes of imaging services identified in the discussions. We also added a table with the demographics of the participants to the Appendix. Per the reviewer’s request we also added the list of questions in the Appendix.

Page 15, line 352 – “Figure 1 shows an example of a choice task in the DCE” – Is this appendix or is this supposed to be in the main paper? If it is appendix, refer it is in appendix. If not, you don’t need to answer this comment. Just a note that the picture is still blurry and should be corrected at some point.

We added in a new version of the Figure

Page 17, line 368 – “Mixed logit results” – I find it strange you present no comments on heterogeneity in this section? Mixed logit models are one of the two major alternatives of dealing with heterogeneity. So, I would expect you to report and comment on heterogeneity in this section. Also, why did you decide to go with both a mixed logit and a latent class model? Were you unsure if unobserved preference followed a continuous or discrete distribution and wanted to test both? If that is the case you should have said that and comment on both results.

The reviewer is correct that Mixed logit models deal with heterogeneity through interactions. The challenge in this study was sample size to model several interactions. We used both approaches in this study to seek some understanding of the relative merits of both modelling strategies, each regarded as an advanced interpretation of discrete choice models. Both models offer alternative ways of capturing unobserved heterogeneity and other potential sources of variability in unobserved sources of utility. We added in a lot of new text to clarify this.

Page 23, line 482 – “relatively rural area in the Northeast” – always write Northeast of the Unite States to clarify.

We added “of the United States”.

Page 23, line 485 – “Most RRCs do not currently offer online scheduling so ease of use may also steer patients towards HORDs.” – I think a comma is needed for the sentence to be easier to read. Also, the “ease of use” expression bugs me a little. I understand what you want to say, but the idea of the RRCs was also to increase access, correct? Maybe it is just me, but I tend to put ease of access in the mix for ease of use. If you agree with, maybe consider changing or clarifying the expression.

We added a comma. We agree and changed “ease of use” to “ease of access”.

Page 23, line 501 to 502 “and what would - “push them over the fence” to seek services elsewhere” – Just highlight that your paper is already providing important information for that type of analysis. Knowing how much people value certain characteristics will inform if services can have enough of those to make people using them, and if they can make it a cost that is inferior to peoples willingness to pay, so they can increase the prices to cover those costs while capturing more demand.

Correct and we agree with the reviewer that’s what we’ve studied. We edited the sentence to clarify that follow-up work should further analyze preference heterogeneity. With a larger sample size, interactions in a mixed logit model could give a better understanding of how trade-offs made differ between patients. The reviewer has also made this point above.

Page 24, line 520 to 522 – “Moreover, based on our qualitative research we used a complex design for the DCE, although NGene showed that we had sufficient sample size for both X-ray and MRI.” – This sentence seems to have parachuted itself into the middle of another paragraph. Please, find another place for it. Also, it still does not comment on one possible limitation of your paper, which is whether it is likely that people suffered from any cognitive burden in your analysis, since 3 choice sets with 10 attributes and 14 choices sets is an extremely complex design.

We moved the sentence to the section where we also address the attention check.

Page 25, line 546 to 549 – “The marginal rates of substitution in terms of costs were shown in table 5; followup work will focus on other trade-offs such as how much travel time patients are willing to trade-off for service or shorter wait time to results. In a national follow-up study, we will collect more data to this analysis.” – You should not refer to a table in the conclusion. You also should not say that some more work is going to be done. Instead, you may say that through your study, further research was identified as needed (but only if this is the case). In general, I don’t like this entire sentence and don’t understand its purpose.

We agree with the reviewer and we have deleted the entire sentence.

Thank you for your comments and suggestions!
