# Supplementary material for: Patient preferences for diagnostic imaging services: Decentralize or not?
Source: PLoS One. 2025 May 16;20(5):e0301404. doi: 10.1371/journal.pone.0301404 (PMC12084043; doi:10.1371/journal.pone.0301404)
Supplement: Attachment — Submitted filename: Response to Reviewers_3_Decentralized Imaging.docx [file pone.0301404.s007.docx]

**Response to Reviewers.**

Thank you for your additional comments. Please find our response to these issues.

In this revision and the previous one, you added new text containing typos or with not optimally structured sentences, or even lacking clarity. Please re-read the bits that were added and adjust whenever needed.
For example, there is a typo at line 195, page 8 (track changes version): “The levels were partly define don concrete contributions”.

We re-read the full manuscript and corrected all typos and sentences that needed to be edited.

Another example, line 314, page 16: “The quotas that were agreed on included 5 percent of census on age by decade, gender, and region; 3 percent of census on race/ethnicity; and 50 percent above and below national median income of $65k/year.”. It is not very clear what you mean by 5 percent of census on age by decade, gender and region. Shouldn’t gender be around 50%/50%, for example?

The percentages refer to the Census method of covering a particular percentage of the total population. We left this out to avoid confusion.

Another one, on line 328, page 17: “We used a complex design for the DCE, although NGene showed that we had sufficient sample size for both X-ray and MRI.” – what is a complex design? Which method does NGene use? Enough sample size for the separate analysis on the X-ray group and MRI group of respondents?

We did mean “complex” but a design with many attributes and levels. We created an efficient design with Ngene to reduce the dimensions of the matrix and getting optimal information. A design is considered more efficient if it can produce more efficient data in the sense that more reliable parameter estimates can be achieved with an equal or lower sample size. This process is explained on page 10.

Another one, line 576, page 29: “This could be due to parking being considered a “different kind of expense”, but we would leave out costs for parking out of the design in a next round of data collection.” – Future research is not relevant here. Just state the limitation and move on. Also, I wouldn’t talk about correlation tests because there is an obvious theoretical perfect correlation. Simple example, do you prefer to go to a GP consultation and pay 50 dollars + 10 dollars on parking or pay 60 dollars + 0 dollars on parking? They are the same because you pay 60 dollars anyway. The factor that may be relevant is whether parking is available. It is possible that respondents answered that part with another perspective. For instance, they may have associated free parking with how easy parking would be.

Agreed. We deleted “We did check for correlation and do not find the two coefficients to be correlated. This could be due to parking being considered a “different kind of expense”, but we would leave out costs for parking out of the design in a next round of data collection.”

I would still like to see a comment on how likely it is that your design led to cognitive burden issues, because 14 choice sets with 3 alternatives each and 10 attributes is not easy task. Did you pre-test the design? Did people say it was ok? How long did they take to answer the survey? Please say something to inform the reader on whether this might have been a problem or not.

We added a clarification to the limitations section.

I would still like to see the survey. If you can’t find an easy way to export it, you should find another alternative (print page by page, copy paste question on another document, etc).

We uploaded the html version of the survey text as a supplemental file.
